# 1,1′-[(Hexane-1,6-diyldi­oxy)bis­(nitrilo­methyl­idyne)]dinaphthalene

**DOI:** 10.1107/S160053680801920X

**Published:** 2008-07-05

**Authors:** Wen-Kui Dong, Xue-Ni He, Li Li, Zhong-Wu Lv, Jun-Feng Tong

**Affiliations:** a School of Chemical and Biological Engineering, Lanzhou Jiaotong University, Lanzhou 730070, People’s Republic of China

## Abstract

The title compound, C_28_H_28_N_2_O_2_, was synthesized by condensation of 1-naphthaldehyde with 1,6-bis­(amino­oxy)hexane in ethanol. The mol­ecule is disposed about a crystallographic centre of symmetry. In the crystal structure, mol­ecules are linked through strong inter­molecular π–π stacking inter­actions [interplana distance = 2.986 (2) Å], forming a three-dimensional network.

## Related literature

For related literature, see: Akine *et al.* (2006 ▶); Dong *et al.* (2007 ▶); Herzfeld & Nagy (1999 ▶); Shi *et al.* (2007 ▶); You *et al.* (2004 ▶).
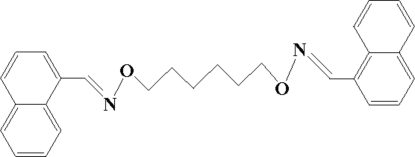


## Experimental

### 

#### Crystal data

C_28_H_28_N_2_O_2_
*M* *_r_* = 424.52
Monoclinic, 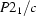

*a* = 9.2925 (16) Å
*b* = 6.3938 (12) Å
*c* = 19.723 (2) Å
β = 96.489 (2)°
*V* = 1164.3 (3) Å^3^
*Z* = 2
Mo *K*α radiation
μ = 0.08 mm^−1^
*T* = 298 (2) K
0.47 × 0.42 × 0.23 mm

#### Data collection

Bruker SMART 1000 CCD area-detector diffractometer
Absorption correction: multi-scan (*SADABS*; Sheldrick, 1996 ▶) *T*_min_ = 0.965, *T*_max_ = 0.983
5470 measured reflections
2050 independent reflections
1047 reflections with *I* > 2σ(*I*)
*R*_int_ = 0.040

#### Refinement

*R*[*F* ^2^ > 2σ(*F* ^2^)] = 0.053
*wR*(*F* ^2^) = 0.154
*S* = 1.07
2050 reflections
145 parameters
H-atom parameters constrained
Δρ_max_ = 0.18 e Å^−3^
Δρ_min_ = −0.12 e Å^−3^

### 

Data collection: *SMART* (Siemens, 1996 ▶); cell refinement: *SAINT* (Siemens, 1996 ▶); data reduction: *SAINT*; program(s) used to solve structure: *SHELXS97* (Sheldrick, 2008 ▶); program(s) used to refine structure: *SHELXL97* (Sheldrick, 2008 ▶); molecular graphics: *SHELXTL* (Sheldrick, 2008 ▶); software used to prepare material for publication: *SHELXTL*.

## Supplementary Material

Crystal structure: contains datablocks global, I. DOI: 10.1107/S160053680801920X/hg2416sup1.cif

Structure factors: contains datablocks I. DOI: 10.1107/S160053680801920X/hg2416Isup2.hkl

Additional supplementary materials:  crystallographic information; 3D view; checkCIF report

## Figures and Tables

**Fig. 1.**
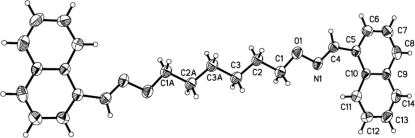
The molecule structure of (I) with atom numbering (symmetry code A: -x + 1, -y, -z). Displacement ellipsoids for non-hydrogen atoms are drawn at the 30% probability level.

**Fig. 2.**
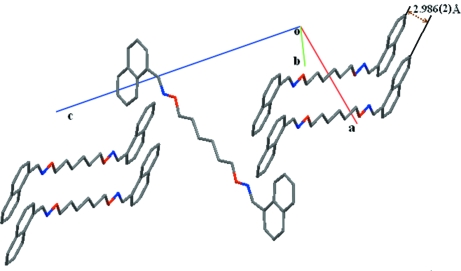
Crystal structure of (I) showing the formation of π–π interactions (Inter-molecular plane-to-plane distance, 2.986 (2) Å).

## supplementary crystallographic information

### Comment

Schiff-base compounds containing imine groups have been used as modulators of structural and electronic properties of transition matal centres in modern coordination chemistry (You, *et al.*, 2004). The diversity in the coordination environment and structures of transition metal complexes mainly depend on the type of Schiff-base ligands (Herzfeld, *et al.*, 1999). In this research, we report on the synthesis and crystal structure of (I) with the aim of confirming its structural properties, and gaining further insight into its coordinating abilities toward various transition metal ions.

The crystal structure of (I) consists of discrete molecules disposed about a crystallographic centre of symmetry. The six carbon atoms in the (—CH=N—O—(CH_2_)_6_—O—N=CH—) bridge deviate slightly from the mean plane, with C1, C2 and C3 above by 0.04, 0.04 and 0.08 Å, and C1A, C2A and C3A below by 0.04, 0.04 and 0.08 Å (symmetry code A: -*x* + 1, -*y*, -*z*), respectively. The planes of the two naphthane rings in (I) are parallel with a separation distance of 2.163 (2) Å. In the crystal structure, molecules are linked through strong intermolecular π–π stacking interactions (Inter-molecular plane-to-plane distance, 2.986 (2) Å) to form a three-dimensional network.

### Experimental

1,1'-[Hexane-1,6-diyldioxybis(nitrilomethylidyne)]dinaphthalene was synthesized according to an analogous method reported earlier (Shi, *et al.*, 2007; Akine, *et al.*, 2006; Dong, *et al.*, 2007). To an ethanol solution (5 ml) of 1-naphthaldehyde (644.1 mg, 4.00 mmol) was added an ethanol solution (5 ml) of 1, 6-bis(aminooxy)hexane (296.5 mg, 2.00 mmol). The mixed solution was stirred at 328 K for 5 h. When cooled to room temperature, the precipitate was filtered, and washed successively with ethanol and hexane, respectively. The product was dried under vacuum and purified with recrystallization from ethanol to yield 637.4 mg of (I). Yield, 75.1%. mp. 348–349 K. Anal. Calc. for C_28_H_28_N_2_O_2_: C, 79.22; H, 6.65; N, 6.60. Found: C, 79.35; H, 6.75; N, 6.53.

Colorless block-shaped single crystals suitable for X-ray diffraction studies were obtained after several weeks by slow evaporation from an methanol solution of (I).

### Refinement

Non-H atoms were refined anisotropically. H atoms were treated as riding atoms with distances C—H = 0.97 (CH_2_), or 0.93 Å (CH), and *U*_iso_(H) = 1.2 *U*_eq_(C) and 1.5 *U*_eq_(O).

### Crystal data

**Table:**

C_28_H_28_N_2_O_2_	*F*_000_ = 452
*M**_r_* = 424.52	*D*_x_ = 1.211 Mg m^−^^3^
Monoclinic, *P*2_1_/*c*	Mo *K*α radiation λ = 0.71073 Å
Hall symbol: -P 2ybc	Cell parameters from 1078 reflections
*a* = 9.2925 (16) Å	θ = 2.2–22.9º
*b* = 6.3938 (12) Å	µ = 0.08 mm^−^^1^
*c* = 19.723 (2) Å	*T* = 298 (2) K
β = 96.489 (2)º	Block-shaped, colorless
*V* = 1164.3 (3) Å^3^	0.47 × 0.42 × 0.23 mm
*Z* = 2	

### Data collection

**Table:**

Bruker SMART 1000 CCD area-detector diffractometer	2050 independent reflections
Radiation source: fine-focus sealed tube	1047 reflections with *I* > 2σ(*I*)
Monochromator: graphite	*R*_int_ = 0.040
*T* = 298(2) K	θ_max_ = 25.0º
φ and ω scans	θ_min_ = 2.1º
Absorption correction: multi-scan(SADABS; Sheldrick, 1996)	*h* = −11→10
*T*_min_ = 0.965, *T*_max_ = 0.983	*k* = −7→3
5470 measured reflections	*l* = −22→23

### Refinement

**Table:**

Refinement on *F*^2^	Secondary atom site location: difference Fourier map
Least-squares matrix: full	Hydrogen site location: inferred from neighbouring sites
*R*[*F*^2^ > 2σ(*F*^2^)] = 0.053	H-atom parameters constrained
*wR*(*F*^2^) = 0.155	*w* = 1/[σ^2^(*F*_o_^2^) + 0.4185*P*] where *P* = (*F*_o_^2^ + 2*F*_c_^2^)/3
*S* = 1.08	(Δ/σ)_max_ < 0.001
2050 reflections	Δρ_max_ = 0.18 e Å^−^^3^
145 parameters	Δρ_min_ = −0.12 e Å^−^^3^
Primary atom site location: structure-invariant direct methods	Extinction correction: none

### Special details

**Table:**

Geometry. All e.s.d.'s (except the e.s.d. in the dihedral angle between two l.s. planes) are estimated using the full covariance matrix. The cell e.s.d.'s are taken into account individually in the estimation of e.s.d.'s in distances, angles and torsion angles; correlations between e.s.d.'s in cell parameters are only used when they are defined by crystal symmetry. An approximate (isotropic) treatment of cell e.s.d.'s is used for estimating e.s.d.'s involving l.s. planes.
Refinement. Refinement of *F*^2^ against ALL reflections. The weighted *R*-factor *wR* and goodness of fit *S* are based on *F*^2^, conventional *R*-factors *R* are based on *F*, with *F* set to zero for negative *F*^2^. The threshold expression of *F*^2^ > σ(*F*^2^) is used only for calculating *R*-factors(gt) *etc*. and is not relevant to the choice of reflections for refinement. *R*-factors based on *F*^2^ are statistically about twice as large as those based on *F*, and *R*- factors based on ALL data will be even larger.

### Fractional atomic coordinates and isotropic or equivalent isotropic displacement parameters (Å^2^)

**Table:**

	*x*	*y*	*z*	*U*_iso_*/*U*_eq_	
N1	0.2373 (3)	0.6843 (4)	0.09525 (11)	0.0619 (7)	
O1	0.2359 (2)	0.5082 (3)	0.05242 (10)	0.0696 (6)	
C1	0.3666 (3)	0.3957 (4)	0.06716 (14)	0.0599 (8)	
H1A	0.3749	0.3439	0.1137	0.072*	
H1B	0.4486	0.4861	0.0624	0.072*	
C2	0.3651 (3)	0.2176 (5)	0.01842 (14)	0.0608 (8)	
H2A	0.3492	0.2722	−0.0277	0.073*	
H2B	0.2840	0.1272	0.0250	0.073*	
C3	0.5006 (3)	0.0891 (4)	0.02526 (13)	0.0633 (9)	
H3A	0.5156	0.0321	0.0711	0.076*	
H3B	0.5820	0.1798	0.0195	0.076*	
C4	0.1146 (4)	0.7770 (5)	0.08649 (13)	0.0580 (8)	
H4	0.0423	0.7156	0.0565	0.070*	
C5	0.0789 (3)	0.9699 (5)	0.11943 (13)	0.0526 (7)	
C6	−0.0446 (3)	1.0682 (5)	0.09222 (15)	0.0658 (9)	
H6	−0.1010	1.0040	0.0562	0.079*	
C7	−0.0904 (4)	1.2583 (6)	0.11549 (17)	0.0755 (10)	
H7	−0.1745	1.3205	0.0947	0.091*	
C8	−0.0126 (4)	1.3520 (5)	0.16820 (17)	0.0721 (10)	
H8	−0.0436	1.4792	0.1842	0.086*	
C9	0.1160 (3)	1.2596 (5)	0.19969 (14)	0.0562 (8)	
C10	0.1637 (3)	1.0647 (5)	0.17612 (13)	0.0498 (7)	
C11	0.2900 (3)	0.9759 (5)	0.21026 (14)	0.0661 (9)	
H11	0.3231	0.8479	0.1959	0.079*	
C12	0.3639 (4)	1.0755 (7)	0.26392 (17)	0.0859 (11)	
H12	0.4470	1.0142	0.2861	0.103*	
C13	0.3182 (5)	1.2668 (7)	0.28634 (18)	0.0911 (12)	
H13	0.3713	1.3338	0.3228	0.109*	
C14	0.1975 (4)	1.3554 (6)	0.25544 (18)	0.0798 (11)	
H14	0.1672	1.4832	0.2713	0.096*	

### Atomic displacement parameters (Å^2^)

**Table:**

	*U*^11^	*U*^22^	*U*^33^	*U*^12^	*U*^13^	*U*^23^
N1	0.0736 (19)	0.0517 (16)	0.0629 (16)	−0.0071 (14)	0.0189 (13)	−0.0125 (13)
O1	0.0779 (15)	0.0602 (14)	0.0711 (13)	0.0002 (12)	0.0109 (11)	−0.0154 (12)
C1	0.067 (2)	0.0532 (19)	0.0614 (18)	−0.0088 (17)	0.0143 (15)	−0.0052 (16)
C2	0.071 (2)	0.0524 (18)	0.0598 (18)	−0.0114 (17)	0.0115 (15)	−0.0068 (16)
C3	0.073 (2)	0.056 (2)	0.0618 (18)	−0.0078 (18)	0.0109 (16)	−0.0047 (15)
C4	0.065 (2)	0.057 (2)	0.0525 (17)	−0.0090 (18)	0.0092 (15)	−0.0024 (16)
C5	0.0578 (18)	0.0535 (19)	0.0489 (15)	−0.0051 (16)	0.0166 (14)	0.0034 (15)
C6	0.062 (2)	0.074 (2)	0.0617 (19)	−0.0002 (19)	0.0080 (16)	0.0071 (18)
C7	0.067 (2)	0.080 (3)	0.080 (2)	0.011 (2)	0.0131 (19)	0.010 (2)
C8	0.079 (2)	0.061 (2)	0.083 (2)	0.007 (2)	0.039 (2)	0.007 (2)
C9	0.062 (2)	0.059 (2)	0.0526 (17)	−0.0102 (18)	0.0249 (15)	−0.0031 (16)
C10	0.0535 (18)	0.0528 (19)	0.0455 (15)	−0.0091 (16)	0.0160 (13)	−0.0003 (14)
C11	0.068 (2)	0.069 (2)	0.0614 (18)	0.0025 (18)	0.0087 (16)	−0.0055 (18)
C12	0.071 (2)	0.116 (3)	0.068 (2)	0.001 (2)	−0.0050 (18)	−0.016 (2)
C13	0.087 (3)	0.118 (4)	0.069 (2)	−0.025 (3)	0.010 (2)	−0.037 (2)
C14	0.086 (3)	0.079 (3)	0.080 (2)	−0.014 (2)	0.034 (2)	−0.020 (2)

### Geometric parameters (Å, °)

**Table:**

N1—C4	1.279 (3)	C6—C7	1.383 (4)
N1—O1	1.407 (3)	C6—H6	0.9300
O1—C1	1.413 (3)	C7—C8	1.339 (4)
C1—C2	1.490 (4)	C7—H7	0.9300
C1—H1A	0.9700	C8—C9	1.412 (4)
C1—H1B	0.9700	C8—H8	0.9300
C2—C3	1.497 (4)	C9—C14	1.404 (4)
C2—H2A	0.9700	C9—C10	1.419 (4)
C2—H2B	0.9700	C10—C11	1.406 (4)
C3—C3^i^	1.513 (5)	C11—C12	1.354 (4)
C3—H3A	0.9700	C11—H11	0.9300
C3—H3B	0.9700	C12—C13	1.383 (5)
C4—C5	1.450 (4)	C12—H12	0.9300
C4—H4	0.9300	C13—C14	1.340 (5)
C5—C6	1.364 (4)	C13—H13	0.9300
C5—C10	1.428 (4)	C14—H14	0.9300
			
C4—N1—O1	110.0 (2)	C5—C6—H6	118.3
N1—O1—C1	109.6 (2)	C7—C6—H6	118.3
O1—C1—C2	108.2 (2)	C8—C7—C6	119.4 (3)
O1—C1—H1A	110.1	C8—C7—H7	120.3
C2—C1—H1A	110.1	C6—C7—H7	120.3
O1—C1—H1B	110.1	C7—C8—C9	120.7 (3)
C2—C1—H1B	110.1	C7—C8—H8	119.7
H1A—C1—H1B	108.4	C9—C8—H8	119.7
C1—C2—C3	114.5 (2)	C14—C9—C8	121.1 (3)
C1—C2—H2A	108.6	C14—C9—C10	118.6 (3)
C3—C2—H2A	108.6	C8—C9—C10	120.2 (3)
C1—C2—H2B	108.6	C11—C10—C9	118.2 (3)
C3—C2—H2B	108.6	C11—C10—C5	124.1 (3)
H2A—C2—H2B	107.6	C9—C10—C5	117.7 (3)
C2—C3—C3^i^	114.2 (3)	C12—C11—C10	120.5 (3)
C2—C3—H3A	108.7	C12—C11—H11	119.8
C3^i^—C3—H3A	108.7	C10—C11—H11	119.8
C2—C3—H3B	108.7	C11—C12—C13	121.2 (3)
C3^i^—C3—H3B	108.7	C11—C12—H12	119.4
H3A—C3—H3B	107.6	C13—C12—H12	119.4
N1—C4—C5	125.4 (3)	C14—C13—C12	120.0 (3)
N1—C4—H4	117.3	C14—C13—H13	120.0
C5—C4—H4	117.3	C12—C13—H13	120.0
C6—C5—C10	118.5 (3)	C13—C14—C9	121.4 (3)
C6—C5—C4	116.1 (3)	C13—C14—H14	119.3
C10—C5—C4	125.3 (3)	C9—C14—H14	119.3
C5—C6—C7	123.4 (3)		
			
C4—N1—O1—C1	−174.4 (2)	C8—C9—C10—C11	−178.2 (3)
N1—O1—C1—C2	−176.9 (2)	C14—C9—C10—C5	179.9 (2)
O1—C1—C2—C3	177.0 (2)	C8—C9—C10—C5	1.1 (4)
C1—C2—C3—C3^i^	−178.9 (3)	C6—C5—C10—C11	177.6 (3)
O1—N1—C4—C5	−176.7 (2)	C4—C5—C10—C11	−3.9 (4)
N1—C4—C5—C6	165.6 (3)	C6—C5—C10—C9	−1.7 (4)
N1—C4—C5—C10	−13.0 (4)	C4—C5—C10—C9	176.8 (2)
C10—C5—C6—C7	1.8 (4)	C9—C10—C11—C12	−0.3 (4)
C4—C5—C6—C7	−176.8 (3)	C5—C10—C11—C12	−179.6 (3)
C5—C6—C7—C8	−1.3 (5)	C10—C11—C12—C13	−0.4 (5)
C6—C7—C8—C9	0.6 (5)	C11—C12—C13—C14	1.0 (5)
C7—C8—C9—C14	−179.3 (3)	C12—C13—C14—C9	−0.8 (5)
C7—C8—C9—C10	−0.6 (4)	C8—C9—C14—C13	178.8 (3)
C14—C9—C10—C11	0.5 (4)	C10—C9—C14—C13	0.0 (5)

Symmetry codes: (i) −*x*+1, −*y*, −*z*.
